# SARS-CoV-2 reinfection and COVID-19 severity

**DOI:** 10.1080/22221751.2022.2052358

**Published:** 2022-03-21

**Authors:** Nhu Ngoc Nguyen, Linda Houhamdi, Van Thuan Hoang, Jeremy Delerce, Léa Delorme, Philippe Colson, Philippe Brouqui, Pierre-Edouard Fournier, Didier Raoult, Philippe Gautret

**Affiliations:** a IRD, AP-HM, SSA, VITROME, Aix Marseille University, Marseille, France; b IHU-Méditerranée Infection, Marseille, France; c Thai Binh University of Medicine and Pharmacy, Thai Binh, Vietnam; d IRD, AP-HM, MEPHI, Aix-Marseille University, Marseille, France

**Keywords:** SARS-CoV-2, COVID-19, coronavirus, reinfection, severity

## Abstract

SARS-CoV-2 reinfection rate is low. The relative severity of the first and second episodes of infection remains poorly studied. In this study, we aimed at assessing the frequency of SARS-CoV-2 reinfections and comparing the severity of the first and second episodes of infection. We retrospectively included patients with SARS-CoV-2 positive RT-PCR at least 90 days after clinical recovery from a COVID-19 episode and with at least one negative RT-PCR after the first infection. Whole genome sequencing and variant-specific RT-PCR were performed and clinical symptoms and severity of infection were retrospectively documented from medical files. A total of 209 COVID-19 reinfected patients were identified, accounting for 0.4% of positive cases diagnosed from 19 March 2020 to 24 August 2021. Serology was performed in 64 patients, of whom 39 (60.1%) had antibodies against SARS-CoV-2 when sampled at the early stage of their second infection. Only seven patients (3.4%) were infected twice with the same variant. We observed no differences in clinical presentation, hospitalization rate, and transfer to ICU when comparing the two episodes of infections. Our results suggest that the severity of the second episode of COVID-19 is in the same range as that of the first infection, including patients with antibodies.

## Introduction

COVID-19, caused by the Severe Acute Respiratory Syndrome Virus-2 (SARS-CoV-2), rapidly spread worldwide after the first case was identified in Wuhan, China, in 2019. Nearly two years later, this disease still impacts public health and attracts attention from researchers. According to the WHO, antibody presence in recovered patients does not guarantee protection from reinfection, evaluated at 50% for patients aged more than 65 years old [1]. In 2020, the first observed SARS-CoV-2 reinfection cases were reported in Hong Kong, with 142 days between two episodes, with mild symptoms for the first and no symptoms for the second infection [2]. A large prospective cohort study conducted in England on 25,611 individuals showed that a person with a history of previous SARS-CoV-2 infection has an 84% lower risk of reinfection during the seven months following a primary infection as compared to naïve patients [3]. In a study conducted among 829 patients recovered from COVID-19, 87 had no detectable IgG against SARS-CoV-2, of whom 25 (28.7%) were reinfected 1–3 months after their first infection, while there was just one case of reinfection 4.5 months after initial recovery among those with detectable IgG (0.1%) [4]. Recently, a meta-analysis showed that the pooled estimation of reinfection among recovered patients was 0.3%, with a high heterogeneity among studies, and it was more common among male patients [5].

Depending on the epidemic periods, the proportion of reinfection was at 0.61% and 0.08% in patients seen at our institute and primarily infected during the first (from February 2020 to May 2020) and second waves (from mid-June 2020 to February 2021), respectively [6]. In a preliminary study conducted in our centre in 46 reinfected COVID-19 patients, the proportion of patients with severe/critical status was significantly higher during the second episode than the first (21.2% vs. 5.1%); however, the hospitalization rate, transfer to intensive care unit and lethality did not differ between the two episodes of infection [7].

In this work, we aimed at assessing the frequency of SARS-CoV-2 reinfections and at comparing the severity of the first infection and reinfection over a longer period of study.

## Materials and methods

### Study designs and data collection

From 19 March 2020 to 24 August 2021, 506,238 persons presented at IHU Méditerranée Infection for SARS-CoV-2 testing. Of whom, 777,437 nasopharyngeal samples were collected and screened by RT-PCR test. The IHU Méditerranée Infection is the largest specialized structure in the Marseille area (the second largest town in France with 1,620,227 inhabitants [8]). It comprises 75 beds in three wards and a large outpatient department that were dedicated to COVID-19 patient management since the start of the epidemic. It also comprises a large laboratory serving the entire Assistance Publique–Hôpitaux de Marseille (APHM) including COVID-19 temporary wards and intensive care units. Samples from patients handled outside of these structures are also sent by many peripheral laboratories in the region. Patients with RT-PCR-confirmed SARS-CoV-2 infection diagnosed at the laboratory of our institute were retrospectively screened for possible reinfection.

According to the CDC definition, reinfection in this study was defined by a positive RT-PCR test at least 90 days after clinical recovery from a first episode and at least one negative RT-PCR after the first infection [9]. A computerized alert system identified reinfected patients based on these criteria. Information on demographics, chronic conditions, vaccination status, serological status, clinical symptoms, and outcomes [hospitalization, transfer to intensive care unit (ICU), and death] during both episodes were retrieved from electronic medical files when available. In addition, we retrieved from medical files information on the presence of antibodies to SARS-CoV-2 in these patients in the early days of the second infection when possible, as assessed by an electrochemiluminescence test (Roche^®^ Diagnostics, Mannheim, Germany) and an immunoenzymatic assay LIAISON^®^ SARS-CoV-2 S1/S2 IgG assay (DiaSorin^®^, Saluggia, Italy). These two techniques were used to measure the exact titre of antibodies present in the patients sera, which were produced against the nucleocapsid (positive when superior to one unit) and spike protein (positive when equal or superior to 15 UA/mL) of SARS-CoV-2, respectively.

### SARS-CoV-2 RNA genotyping

Genotyping was performed with genome sequencing or RT-PCR. SARS-CoV-2 RNA extraction from −80°C-preserved nasopharyngeal swabs was performed using the MagMax™ Viral/Pathogen kit (Thermo Fisher Scientific^®^, Woodward St. Austin, USA) and Kingfisher Flex^®^ System instrument (Thermo Fisher Scientific^®^), according to the manufacturer's instructions. Then, whole generation sequencing (WGS) was performed using the Illumina^®^ COVIDSeq™ Test kit (Illumina® Inc., San Diego, CA, USA) and IDT^®^ PCR Indexes Sets 1–4 (Illumina^®^), according to the manufacturer's instructions. The pool and denaturation of libraries were performed using protocol B of the “NovaSeq 6000 System Denature and Dilute Libraries Guide” (Document #1000000106351 v03, Illumina^®^). The sequencing reaction was run on a NovaSeq™ 6000 instrument (Illumina^®^) for 15  hours.

When WGS was unable to completely genotype the variant, mainly when Cycle threshold (*C*_t_) values were higher than 30, SARS-CoV-2 genotyping was performed using RT-PCR systems that screened the most frequent viral variants circulating in France, depending on the date of the infection [10]. The detailed strategy has been described elsewhere [7,10,11].

### Genome sequence analyses

Consensus genomes were generated by mapping on the Wuhan-Hu-1 isolate genome (GenBank accession No. NC_045512.2) with the CLC Genomics workbench v.7 (https://digitalinsights.qiagen.com/) or the Minimap2 software [12].

Sequences described in the present study have been deposited in the GISAID sequence database (https://www.gisaid.org/) [13] and the IHU Marseille Infection website: https://www.mediterranee-infection.com/tout-sur-le-coronavirus/sequencage-genomique-sars-cov-2/. A phylogenetic tree was built with the Nextstrain/ncov tool (https://github.com/nextstrain/ncov) [14] that performs maximum-likelihood phylogeny using IQ-TREE [15] and then visualized with Auspice (https://docs.nextstrain.org/projects/auspice/en/stable/).

### Data analysis

Data analyses were performed using the Stata 14.2 software (StataCorp LP, College Station, USA) and the R 3.6.2 software (R Foundation for Statistical Computing, Vienna, Austria).

Categories and continuous variables are presented in numbers, percentages, and means ± standard deviations (SD), respectively. The Chi-squared test and McNemar test were used to compare the differences in proportions when appropriate.

Clinical symptoms and severity during first and second COVID-19 episodes were compared in each patient and a logistic regression model was applied to compare the severity of the COVID-19 disease of the first infection versus that of the second infection. Because too few patients were transferred to the intensive care unit (ICU), this outcome was not analyzed. We included all variables with a *p*-value lower than 0.2 in the univariate in the multivariate analysis. The best prediction model for risk factors was the lowest values of the Akaike Information Criterion (AIC). We also excluded variables missing more than 5% data from multivariate analysis. A *p*-value <0.05 was considered statically significant.

In addition, for patients reinfected with certain variants of SARS-CoV-2, we selected control patients matched by age, sex, and comorbidities and experiencing a primary infection with the same variants. In these two sets of patients, disease severity was compared.

### Ethical approval

This retrospective study has been approved by the ethics committee of our institute (No. 2020-016-03). Access to the patients’ biological and registry data issued from the hospital information system was approved by the data protection committee of Assistance Publique-Hôpitaux de Marseille and was recorded in the European General Data Protection Regulation registry under number RGPD/APHM 2019-73, RGPD/APHM 2020-150, RGPD/APHM 2020-151 and RGPD/APHM nov-20 2020-152.

## Results

In this study, from 19 March 2020 to 24 August 2021, we identified a total of 209 patients who had undergone two successive SARS-CoV-2 infection episodes corresponding to CDC COVID-19 reinfection criteria. A flowchart of patient selection is shown in Supplementary Figure 1. Of these patients, 121 had clinical data available for the two episodes of infection.

### Distribution of reinfection cases over time

**Figure 1.**
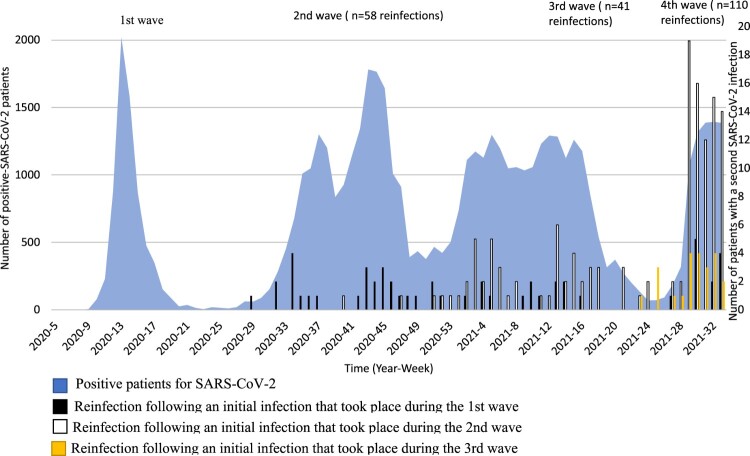
Dynamic of SARS-CoV-2 infections and reinfections diagnosed at IHU Méditeranée Infection, 2020–2021.

From 19 March 2020 to 24 August 2021, based on our laboratory database we diagnosed 55,338 cases of COVID-19, and the epidemic evolved in four waves (Figure 1). The first, from February to early June 2020, was due to three major clades, including 20A (B.1), 20B (B.1.1), and 20C (B.1). The second wave that took place between mid-June 2020 and February 2021 was linked to 20 A (B.1.416) and 20A.EU2 (B.1.160) variants. The third wave occurred from March 2021 to the end of June 2021 and was due to a variant harbouring the N501Y mutation and the 20I (Alpha.V1) (B.1.1.7) variant. Finally, the fourth phase of the epidemic, starting from July 2021 and still ongoing as of 24 August 2021, was caused by the 21A (Delta) (B.1.617.2) variant.

Most first infections occurred in March and summer 2020, while second infections peaked in summer 2021.

**Figure 2.**
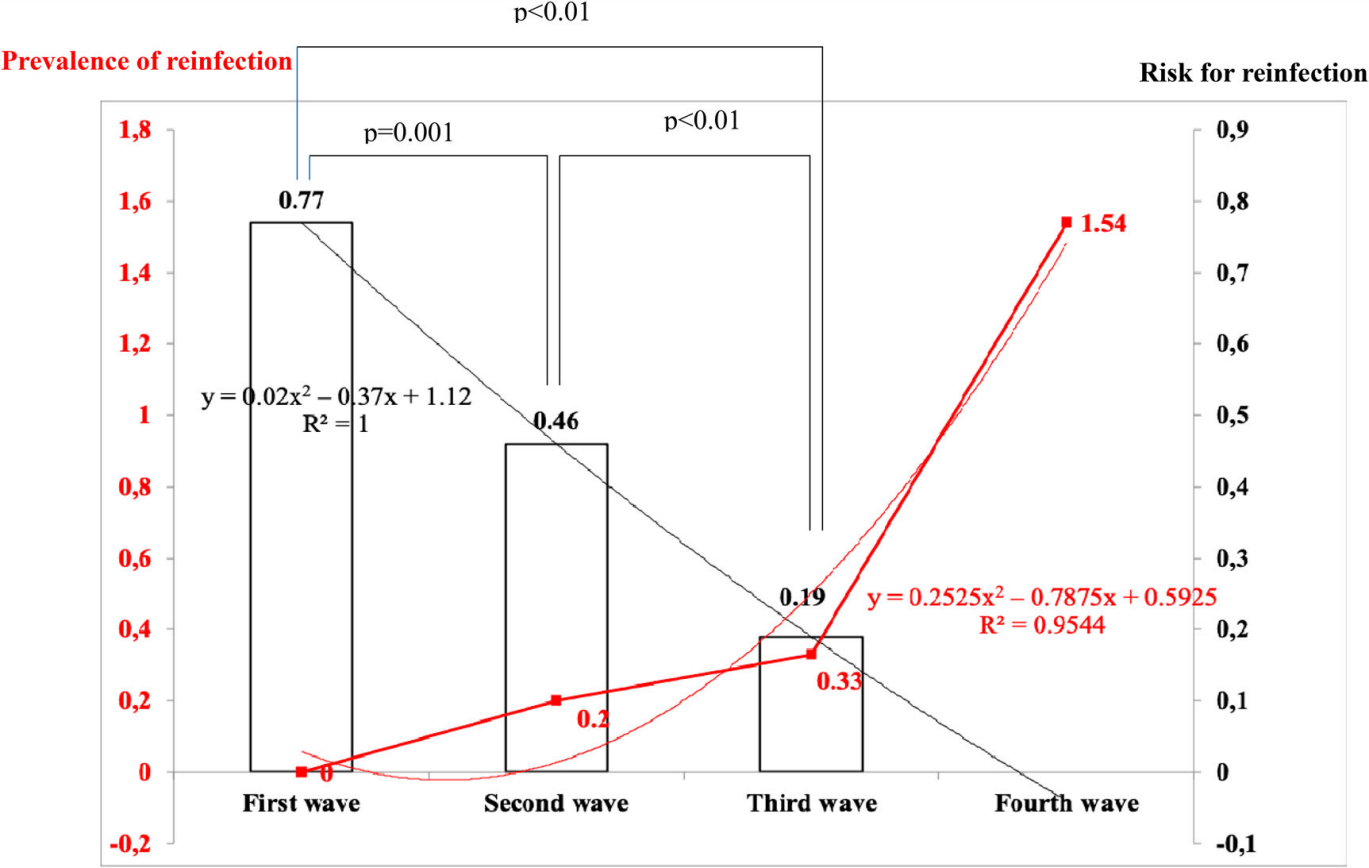
Prevalence of reinfection (proportion of COVID patients who sustained a previous infection with SARS-CoV-2, red curve) and estimated risk for reinfection (proportion of patient infection during a given wave of COVID-19 who got reinfected at the time of study, histograms). The prevalence of reinfection increased from 0 to 1.54% from the first to the fourth wave. The risk for reinfection decreased from 0.77 for the first wave to 0.19 for the third wave (data were not available at the time the study was done to calculate the risk for reinfection for the fourth wave).

Fifty-eight cases of the second infection were observed during the second wave, representing 0.20% of the 29,154 COVID-19 cases diagnosed during the second wave (Figure 2). Forty-one cases were observed during the third wave, accounting for 0.33% of the 12,283 cases diagnosed during the third wave. From July to 24 August 2021, 110 reinfections were observed among the 7152 cases diagnosed during this period of time (1.54%). The prevalence of reinfection significantly increased over time.

Specifically, 52 patients of the 6749 (0.77%) whose first infection took place during the first wave of the epidemic sustained a reinfection. One hundred and thirty-four (134) of the 29,154 (0.46%) patients whose first infection took place during the second wave were reinfected and 23 of the 12,283 (0.19%) whose first infection took place during the third wave were reinfected. Differences in proportion were statistically significant and significantly decreased over time (Figure 2).

### Characteristics of reinfected patients

The baseline characteristics of these 209 patients are detailed in Table 1. In brief, the mean age (± SD) was 40.4 (± 19.8 years) and 51.7% were female. Sixty-eight (68) (38.4%) patients reported at least one comorbidity, with chronic respiratory disease and chronic heart disease the most frequent. Only 6.4% of patients received a vaccine against COVID-19 following their first infection. Serology was performed in 64 patients, of whom 39 (60.1%) had antibodies against SARS-CoV-2 when sampled at the early stage of their second infection. Using the spike – DiaSorin^®^ technique, 33/39 patients were positive with IgG antibody titres varying from 15 to > 400 UA/mL. Of these 33 patients, 11 had a low titre (from 15 to 33 UA/mL). Using the nucleocapsid Roche^®^ technique we identified six patients with antibodies titre from 2 to 190 units. Only one patient had a low titre (two units), while the other five patients had higher titres (from 37 to 190 units). The mean time between two COVID-19 episodes was about seven to eight months, culminating to 17 months in one patient. One-third of patients were reinfected less than six months after the first episode of COVID-19 took place.

**Table 1.** Characteristics of the study population (*N* = 209).

	*N* = 209 (%)
Age (first infection)	
Mean ± SD	40.4 ± 19.8
Range	1–94
<60	177 (84.7)
≥60	32 (15.3)
Gender	
Female	108 (51.7)
Male	101 (48.3)
Comorbidities*^N^*^ = 177^	
At least one condition	68 (38.4)
Chronic respiratory disease	26 (14.7)
Chronic heart disease	23 (13.0)
Hypertension	20 (11.3)
Obesity	20 (11.3)
Diabetes	12 (6.8)
Immunosuppression	5 (2.8)
Vaccination status	
Vaccination (first infection)*^N^*^ = 207^	0 (0.0)
Vaccination (second infection)*^N^*^ = 202^	13 (6.4)
One dose: 0/13 patients	
Two doses: 13/13 patients	
+ Interval between the last dose of vaccine and infection (days)	
Mean ± SD	78.77 ± 49.77
Range	15–153
Antibody present (at second infection) ^*N*= 64^	41 (62.1)
+4/5 vaccinated patients	
+35/59 non-vaccinated patients	
Interval between two infections (days)	
Mean ± SD	232.93 ± 104.02
Range	90–513
≥180 days	130 (62.2)

### Clinical presentation, severity of infection and SARS-CoV-2 variants involved

In these 209 patients, we observed no differences in hospitalization rates and transfer to ICU when comparing the two episodes of infections (Table 2).

**Table 2.** COVID-19 severity and SARS-CoV-2 variants in 209 reinfected patients.

	First infection *N* = 209 (%)	Second infection *N* = 209 (%)	*p*-value*
Hospitalization	19 (9.1%)	13 (6.2%)	0.23
ICU	5 (2.4%)	6 (2.9%)	0.74
Death	NA	2 (1.0%)	NA
**Variant^ ^= 127/167**			
20A.EU2 (B.1.160)	48 (23.0%)	34 (16.3%)	<0.0001
20A (B.1)	25 (12.0%)	0 (0.0%)	
20I (Alpha.V1) (B.1.1.7)	19 (9.1%)	25 (11.9%)	
21A (Delta) (B.1.617.2)	0 (0.0%)	100 (47.9%)	
20C (B.1)	11 (5.2%)	0 (0.0%)	
Others^a^	24 (11.5%)	8 (3.8%)	
Not identified	82 (39.2%)	42 (20.1%)	
**Characteristics of patients with clinical information available**			
	*n* = 121	*n* = 121	
Age (at first infection)			
Mean ± SD	42.83 ± 19.13		–
Range	7–94		–
<60	103 (85.1)		–
≥60	18 (14.9)		–
Gender			
Female	62 (51.2)		–
Male	59 (48.8)		–
**Comorbidities**			
Chronic respiratory disease	20 (16.5)		–
Chronic heart disease	18 (14.9)		–
Hypertension	15 (12.4)		–
Obesity	14 (11.6)		–
Diabetes	10 (8.3)		–
Immunosuppression	5 (4.1)		–
**Clinical symptoms**			
Fever	37 (30.6)	39 (32.2)	0.76
Cough	49 (40.5)	49 (40.5)	1.00
Sore throat	2 (1.7)	5 (4.1)	0.26
Rhinitis	23 (19.0)	17 (14.1)	0.18
Anosmia	26 (21.5)	18 (14.9)	0.19
Ageusia	23 (19.0)	17 (14.1)	0.33
Chest pain	16 (13.2)	12 (9.9)	0.39
Dyspnea	20 (16.5)	25 (20.7)	0.34
Asthenia	36 (29.8)	41 (33.9)	0.48
Headache	36 (29.8)	33 (27.3)	0.63
Myalgia	35 (28.9)	30 (24.8)	0.42
Nausea	1 (0.8)	2 (1.7)	0.56
Vomiting	2 (1.7)	3 (2.5)	0.65
Diarrhoea	22 (18.2)	12 (9.9)	0.03
Asymptomatic	28 (23.1)	35 (28.9)	0.32
**COVID-19 severity**			
Hospitalization	13 (10.7)	12 (9.9)	0.76
ICU	5 (4.1)	6 (4.9)	0.74
Death	0 (0.0)	2 (1.7)	0.16

NA: not applicable.

^a^ 19A (B), 19B (A.19), 20A (B.1), 20A/S:98F (B.1.221), 20A (B.1.416.1), 20B (B.1.1), 20B (B.1.1.269), 20B (B.1.1.241), 20C/S:80Y (B.1.367), 20E (EU1) (B.1.177), 20H (Beta.V2) (B.1.351), 21D (Eta) (B.1.525).

*McNemar test.

Among 13 patients hospitalized during their second infection, eight were males, with ages ranging from 44 to 95 years, and two had received two doses of vaccine. All patients reported comorbidities, six were transferred to ICU and two died (Supplementary Table 1). The proportion of each virus variant during the first and second COVID-19 episodes significantly differed, with the 20A.EU2 (B.1.160) variant being predominant during the first infection and the 21A (Delta) (B.1.617.2) variant being predominant during the second episode (Table 2). In patients experiencing a second infection, the 20I (Alpha.V1) (B.1.1.7), 20A.EU2 (B.1.160) and 21A (Delta) (B.1.617.2) variants accounted for more than 95% of viruses which genome was identified. Only seven patients were infected twice with the same variant (Supplementary Table 2). Six of them experienced mild infections and were not hospitalized. One patient was hospitalized during both episodes of infection and was transferred to ICU during the second episode.

Clinical symptoms during both COVID-19 episodes were available in 121 patients. The prevalence of the various symptoms did not significantly differ between the two episodes of infection, with the notable exception of diarrhoea that was significantly more frequent during the first episode as compared to the second (18.2% vs. 9.9%) (Table 2). No significant differences in COVID-19 severity, as assessed by hospitalization rate and transfer to ICU, were observed between the two episodes of infection in this subpopulation of patients.

### Reinfection versus primary infection with the same SARS-CoV-2 variant

To compare the severity of a first and second infection with the most frequent variants [20I (Alpha.V1) (B.1.1.7), 20A.EU2 (B.1.160), and 21A (Delta) (B.1.617.2)], we compared hospitalization rates, transfer to ICU and deaths in patients matched by age, gender, and comorbidities (Supplementary Table 3). Hospitalization rates and transfer to ICU did not significantly differ between the two groups of patients. No deaths were observed.

In univariate analysis, the risk of hospitalization was significantly higher in patients with older age (≥60 years old) and in those reporting hypertension, diabetes, chronic respiratory disease, chronic heart disease, and obesity. In multivariate analysis, we found that only older age (≥60 years old) remained associated with hospitalization risk (Table 3). Risk factors for transfer to ICU were not investigated because of too small numbers.

**Table 3.** Risk factors for hospitalization among reinfected and primo-infected patients with 20I (Alpha.V1) (B.1.1.7), 20A.EU2 (B.1.160) and 21A (Delta) (B.1.617.2) (paired by age, gender, comorbidities, and variants of SARS-CoV-2) (*N* = 159).

Hospitalization *N* = 318	Univariate analysis	Multivariate analysis
	OR [95%CI] *p*-value	OR [95%CI] *p*-value
Primary infection	ref	
Reinfection	0.79 [0.15–3.77] 0.74	
Age (years)		
<60	Ref	ref
≥60	18.6 [3.68–118.45] <0.0001	10.64 [2.01–56.32] 0.005
Gender		
Female	Ref	
Male	3.96 [0.74–39.49] 0.07	
Comorbidities^N = 289^		
Hypertension		
No	Ref	
Yes	7.74 [1.14–39.59] 0.002	
Diabetes		
No	Ref	
Yes	17.00 [2.27–96.48] <0.0001	
Chronic respiratory disease		
No	Ref	
Yes	4.01 [0.61–19.82] 0.04	
Chronic heart disease		
No	Ref	
Yes	12.23 [1.71–65.70] 0.0001	
Obesity		
No	Ref	
Yes	5.43 [0.50–31.99] 0.03	
Variant		
20I (Alpha.V1) (B.1.1.7)	Ref	
20A.EU2 (B.1.160)	3.06 [0.33–28.27] 0.32	
21A (Delta) (B.1.617.2)	1.00 [0.11–9.15] 1.00	

### Genome sequencing

SARS-CoV-2 genomes from 131 patients were available for incorporation in the phylogeny reconstruction, including from both episodes of infections for 39 patients and from one of the two episodes of infections for 92 patients. Otherwise, the SARS-CoV-2 genotype was determined based on partial genome analysis or variant-specific qPCR (Supplementary Figure 2 and Supplementary Table 4).

## Discussion

Published data on the prevalence of SARS-CoV-2 reinfection highlighted their low rates, ranging from 0.1% to 0.65% [5,16–18]. In our study, we found that overall, 0.38% of patients with SARS-CoV-2 infection diagnosed at our institute had sustained a prior infection with this virus. This proportion slightly increased over time to reach 1.54% during the fourth wave of COVID-19 in the Marseille area, which is likely a mechanical effect due to the increase of cumulative numbers of COVID-19 patients over time. This increase could also be due to the introduction of new variants with some degree of antibody escape such as the Delta variant. When estimating the risk for patients with a first infection to get reinfected, we found that it was less than 1%, suggesting a high rate of protection following natural infection with SARS-CoV-2. In a Danish study conducted on 11,068 patients, the protection rate resulting from a first infection was 80.5% and decreased to 47.1% in older patients (>65 years old) [19]. In our work, cases of reinfection were mostly observed in patients younger than 60 years old (84.7%) who may have had more social contacts than older patients. In addition, we found that 60.0% of unvaccinated reinfected patients with available serological results had antibodies against SARS-CoV-2. This proportion rose to 83.3% in those who were vaccinated. Bean *et al.* reported that reinfection occurred in individuals despite the presence of antibodies against SARS-CoV-2 in their sera [17]. In our series, 64 reinfected patients had available serological results; 39 were positive after the first time of infection and 25 were negative. Among these 39 positive patients, 12 (30.8%) had a low titre of antibodies, which might make them more susceptible to reinfection. However, a high titre of antibodies was observed in the 27 other patients (69.2%), which strongly suggests that antibodies might not protect patients from reinfection with SARS-CoV-2. Unfortunately, serological results were not available from non-reinfected patients, and therefore we cannot formally conclude about the protection rate of these antibodies. We found that the risk of reinfection significantly decreased over time. However, this observation should be considered with caution, since it depends on the cumulative number of reinfections that also increases over time. Of note, the risk of reinfection in patients infected during the second wave of COVID-19 in Marseille was 0.08% in our preliminary study [6], while it was 0.46% in the present study due to the occurrence of new cases of reinfection that were diagnosed after our previous assessment.

Interestingly, one-third of patients were reinfected less than 180 days after the first infection. This observation supports early vaccination following COVID-19, which should be proposed at 60–90 days post-infection rather than at 60–180 days post-infection, as recommended by French authorities [20].

In this work, we did not evidence any significant differences of severity between the first and second infections. However, this might be due to small numbers, with notably only 11 patients who were admitted to ICU. Similarly, in a Mexican study conducted on 315 patients, the authors observed no significant difference in hospitalization rates between the first and second infection [21].

Also, the two episodes in each patient were caused by different SARS-CoV-2 variants in most cases and variant pathogenicity is known to be different [22,23]. It is therefore difficult to evaluate the respective roles of the responsible virus variants and the possible effect of a previous infection in terms of protection or potential facilitating effect. Nevertheless, when comparing patients experiencing the first infection to those sustaining a reinfection with a similar SARS-CoV-2 variant, hospitalization rates were similar, and depended on patient age only. Unfortunately, the numbers were too small to allow investigating risks of transfer to ICU and death. Further studies conducted in larger cohorts of patients will be needed to better investigate the severity of SARS-CoV-2 reinfections.

We acknowledge some limitations of our study. First, we were unable to calculate the risk of reinfection for all the patients after recovery for the first time as we did not have the totality of their genotyping results. Second, we used the computerized alert system to identify the reinfection cases, which underestimates the actual number of reinfected patients, especially those who had only one positive RT-PCR in our institution. Since in this study the infections are identified in our clinic, it is possible that there is an underestimation of reinfection from asymptomatic cases, which might remain undetected if the person does not attend the clinic and does not go through serial testing. Third, we did not have clinical information for all symptomatic individuals. Moreover, 35 of 121 patients were asymptomatic in the second time of reinfection (Table 2) and had performed their RT-PCR for other reasons, such as contact case tracing or testing prior to travelling. Nowadays, Omicron is the latest circulating variant of concern of SARS-CoV-2, detected for the first time on November 2021 in Gauteng province, South Africa and spread fast worldwide [24]. This variant was associated with an increased risk for reinfection in recovered patients [25]. Further study conducted at our place where Omicron is now predominant will be of interest.

## Conclusion

Our results suggest that the severity of the second episode of COVID-19 is in the same range as that of the first infection. These observations support vaccination of at-risk individuals in order to reduce the severity of infections, including those who were previously infected with SARS-CoV-2. Vaccination of previously infected patients should be performed no more than 3 months after recovery.

## Supplementary Material

Supplemental Material
