# Supplementary material for: SARS-CoV-2 reinfection and COVID-19 severity
Source: Emerg Microbes Infect. 2022 Mar 21;11(1):894–901. doi: 10.1080/22221751.2022.2052358 (PMC8942490; doi:10.1080/22221751.2022.2052358)
Supplement: Supplemental Material [file TEMI_A_2052358_SM7445.doc]

**Supplementary Table 1:** Characteristics of 13 patients hospitalized during the second infection

| **Patient number** | **Gender** | **Age** | **Transfer to ICU** | **Death** | **Comorbidities** | **Interval between two infections (days)** | **Vaccination** | **Serology results** | **Severity of first infection** | |
| --- | --- | --- | --- | --- | --- | --- | --- | --- | --- | --- |
|  |  |  |  |  |  |  |  |  | **Hospitalization** | **Transfer to ICU** |
| 01 | Female | 45 | Yes | No | Hypertension, obesity | 214 | No | No information | No | No |
| 02 | Male | 71 | No | No | Hypertension, diabetes | 261 | No | No information | Yes | No |
| 03 | Female | 83 | Yes | No | Hypertension,  Chronic heart disease | 308 | No | Positive | No | No |
| 04 | Female | 91 | Yes | Yes | Hypertension, chronic heart disease | 90 | No | No information | No | No |
| 05 | Female | 95 | No | No | Hypertension, chronic heart disease | 314 | First infection: No  Second infection: Yes, 2 doses | No information | No | No |
| 06 | Male | 59 | Yes | No | Hypertension, diabetes, obesity | 132 | No | No information | Yes | No |
| 07 | Male | 68 | No | No | Hypertension, diabetes, chronic respiratory disease, chronic heart disease, obesity | 162 | No | No information | No | No |
| 08 | Male | 78 | Yes | Yes | Hypertension, chronic heart disease, diabetes | 145 | No | No information | No | No |
| 09 | Male | 62 | No | No | Hypertension, diabetes, chronic respiratory disease, chronic heart disease, obesity | 99 | No | Positive | No | No |
| 10 | Male | 55 | No | No | Hodgkin lymphoma | 225 | First infection: No  Second infection: 2 doses | Positive | Yes | No |
| 11 | Male | 89 | No | No | Hypertension, diabetes | 96 | No | No information | No | No |
| 12 | Female | 44 | No | No | Hypertension, diabetes, chronic heart disease, obesity | 116 | No | Positive | No | No |
| 13 | Male | 79 | Yes | No | Chronic respiratory disease, chronic heart disease | 310 | No | Positive | No | No |

**Supplementary table 2:** Characteristics of 7 patients with the same variants during the two episodes of infection

| **Patient number** | **Gender** | **Age**  **(Second infection)** | **Hospitalization** | **Transfer to ICU** | **Death** | **Comorbidities** | **Interval between two infections (days)** | **Vaccination** | **Serology results** | **Severity of first infection** | |
| --- | --- | --- | --- | --- | --- | --- | --- | --- | --- | --- | --- |
|  |  |  |  |  |  |  |  |  |  | Hospitalization | Transfer to ICU |
| **20I (Alpha.V1) (B.1.1.7) Variant** | | | | | | | | | | | |
| 01 | Female | 28 | No | No | No | No | 134 | No | No information | No | No |
| **20A.EU2 (B.1.160)** | | | | | | | | | | | |
| 02 | Female | 21 | No | No | No | Asthma, smoking | 163 | No | Positive | No | No |
| 03 | Female | 25 | No | No | No | No | 158 | No | Positive | No | No |
| 04 | Male | 44 | No | No | No | HIV | 129 | No | No information | No | No |
| 05 | Male | 56 | No | No | No | No | 170 | No | Negative | No | No |
| 06 | Male | 61 | No | No | No | Pulmonary tuberculosis in 1981, depressive syndrome, obstructive sleep apnea syndrome | 145 | No | No information | No | No |
| 07 | Male | 55 | Yes | No | No | Hodgkin Lymphoma | 225 | First infection: No  Second Infection: two doses | Positive | Yes | Yes |

**Supplementary Table 3.** Comparison of COVID-19 severity in patients matched by age, gender, comorbidities, and SARS-CoV-2 genetic variant and experiencing primary infection or a reinfection with 20I (Alpha.V1) (B.1.1.7), 20A.EU2 (B.1.160) and delta variants 21A (Delta) (B.1.617.2)

|  |  | **Primary infection**  **(n=159)** | **Reinfection***  **(n=159)** | **p-value**  **(chi-square)** |
| --- | --- | --- | --- | --- |
| Age  (years) | Mean ± SD | 37.14± 17.72 | 37.20 ± 17.65 |  |
|  | Range | 4-93 | 4-93 |  |
|  | <60 | 141  (88.7) | 141  (88.7) | 1.00 |
|  | ≥ 60 | 18  (11.3) | 18  (11.3) |  |
| Gender | Female | 83  (52.2) | 83  (52.2) | 1.00 |
|  | Male | 76  (47.8) | 76  (47.8) |  |
| **Comorbidities ^N=159, N=130^** | | | | |
| Hypertension | No | 148  (93.1) | 121  (93.1) | 0.99 |
|  | Yes | 11  (6.9) | 9  (6.9) |  |
| Immunosuppression | No | 158  (99.4) | 129  (99.2) | 1.00 |
|  | Yes | 1  (0.6) | 1  (0.8) |  |
| Diabetes | No | 153  (96.2) | 125  (96.1) | 1.00 |
|  | Yes | 6  (3.8) | 5  (3.9) |  |
| Chronic respiratory disease | No | 142  (89.3) | 113  (86.9) | 0.53 |
|  | Yes | 17  (10.7) | 17  (13.1) |  |
| Chronic heart disease | No | 154  (96.9) | 121  (95.2) | 0.14 |
|  | Yes | 5  (3.1) | 9  (4.8) |  |
| Obesity | No | 151  (95.0) | 122  (93.9) | 0.68 |
|  | Yes | 8  (5.0) | 8  (6.1) |  |
| **Variant** | 20I (Alpha.V1) (B.1.1.7) | 25  (15.7) | 25  (15.7) | 1.00 |
|  | 20A.EU2 (B.1.160) | 34  (21.4) | 34  (21.4) |  |
|  | 21A (Delta) (B.1.617.2) | 100  (62.9) | 100  (62.9) |  |
| **Outcomes** |  |  |  |  |
| Hospitalization | No | 154  (96.9) | 155  (97.5) | 1.00 |
|  | Yes | 5  (3.1) | 4  (2.5) |  |
| ICU | No | 158  (99.4) | 158  (99.4) | 1.00 |
|  | Yes | 1  (0.6) | 1  (0.6) |  |
| Death | No | 159  (100.0) | 159  (100.0) | NA |
|  | Yes | 0 (0.0) | 0 (0.0) |  |

**Supplementary Table 4.** List of genomes incorporated in the phylogenetic analysis

| Patient_Id | Infection: A=1st; B= 2nd | Sampling_date | Sample_Id |
| --- | --- | --- | --- |
| 1 | A | 2020-09-08 | IHUCOVID-33686 |
| 3 | B | 2021-08-23 | IHUCOVID-31622 |
| 4 | A | 2020-08-04 | IHUCOVID-32936 |
| 5 | A | 2020-10-20 | IHUCOVID-32302 |
| 5 | B | 2021-08-24 | IHUCOVID-32334 |
| 6 | B | 2021-08-23 | IHUCOVID-31707 |
| 7 | A | 2020-08-18 | IHUCOVID-1295 |
| 7 | B | 2021-08-22 | IHUCOVID-31613 |
| 8 | A | 2020-09-01 | IHUCOVID-32937 |
| 8 | B | 2021-08-22 | IHUCOVID-31612 |
| 9 | A | 2020-04-01 | IHUCOVID-13301 |
| 10 | A | 2021-03-18 | IHUCOVID-32306 |
| 11 | A | 2021-02-06 | IHUCOVID-32304 |
| 12 | A | 2021-03-23 | IHUCOVID-13740 |
| 13 | B | 2021-08-19 | IHUCOVID-30411 |
| 14 | A | 2020-03-25 | IHUCOVID-0169 |
| 16 | A | 2020-03-27 | IHUCOVID-33682 |
| 17 | B | 2021-08-18 | IHUCOVID-30366 |
| 19 | A | 2021-02-12 | IHUCOVID-25514 |
| 20 | A | 2020-08-24 | IHUCOVID-28685 |
| 21 | B | 2021-08-15 | IHUCOVID-28150 |
| 22 | A | 2021-03-04 | IHUCOVID-30204 |
| 22 | B | 2021-08-14 | IHUCOVID-28135 |
| 23 | A | 2020-11-24 | IHUCOVID-28663 |
| 23 | B | 2021-08-14 | IHUCOVID-28137 |
| 24 | A | 2021-03-04 | IHUCOVID-28000 |
| 24 | B | 2021-08-14 | IHUCOVID-28134 |
| 25 | A | 2020-11-22 | IHUCOVID-28662 |
| 25 | B | 2021-08-14 | IHUCOVID-28136 |
| 26 | B | 2021-08-14 | IHUCOVID-28139 |
| 27 | A | 2020-07-29 | IHUCOVID-28683 |
| 27 | B | 2021-08-13 | IHUCOVID-28567 |
| 28 | B | 2021-08-13 | IHUCOVID-28081 |
| 29 | B | 2021-08-13 | IHUCOVID-28080 |
| 30 | A | 2020-08-06 | IHUCOVID-1116 |
| 31 | B | 2021-08-12 | IHUCOVID-28528 |
| 32 | A | 2021-02-08 | IHUCOVID-24848 |
| 32 | B | 2021-08-12 | IHUCOVID-28498 |
| 33 | A | 2020-04-03 | IHUCOVID-33683 |
| 34 | B | 2021-08-11 | IHUCOVID-33700 |
| 36 | A | 2021-02-16 | IHUCOVID-20746 |
| 36 | B | 2021-08-11 | IHUCOVID-27391 |
| 37 | A | 2021-04-14 | IHUCOVID-28011 |
| 37 | B | 2021-08-10 | IHUCOVID-27945 |
| 38 | A | 2021-02-01 | IHUCOVID-27999 |
| 38 | B | 2021-08-10 | IHUCOVID-27332 |
| 39 | A | 2021-03-22 | IHUCOVID-23754 |
| 39 | B | 2021-08-10 | IHUCOVID-27940 |
| 40 | A | 2021-02-12 | IHUCOVID-27243 |
| 41 | A | 2020-11-16 | IHUCOVID-27973 |
| 43 | A | 2021-01-27 | IHUCOVID-27242 |
| 43 | B | 2021-08-05 | IHUCOVID-27200 |
| 45 | A | 2021-03-17 | IHUCOVID-4812 |
| 46 | B | 2021-08-04 | IHUCOVID-26694 |
| 47 | A | 2020-11-04 | IHUCOVID-33689 |
| 47 | B | 2021-08-03 | IHUCOVID-26690 |
| 48 | A | 2021-04-06 | IHUCOVID-33695 |
| 48 | B | 2021-08-03 | IHUCOVID-27119 |
| 49 | B | 2021-08-03 | IHUCOVID-27112 |
| 50 | A | 2020-09-16 | IHUCOVID-27975 |
| 50 | B | 2021-08-03 | IHUCOVID-27111 |
| 51 | A | 2020-10-07 | IHUCOVID-2314 |
| 51 | B | 2021-08-03 | IHUCOVID-27090 |
| 52 | B | 2021-08-03 | IHUCOVID-27068 |
| 55 | A | 2020-09-01 | IHUCOVID-27241 |
| 55 | B | 2021-08-02 | IHUCOVID-26613 |
| 56 | B | 2021-08-02 | IHUCOVID-26072 |
| 57 | B | 2021-08-01 | IHUCOVID-26058 |
| 58 | A | 2021-01-27 | IHUCOVID-25936 |
| 58 | B | 2021-08-01 | IHUCOVID-23052 |
| 59 | A | 2020-12-14 | IHUCOVID-26654 |
| 59 | B | 2021-07-31 | IHUCOVID-26054 |
| 60 | A | 2020-08-20 | IHUCOVID-26656 |
| 60 | B | 2021-07-31 | IHUCOVID-26016 |
| 61 | B | 2021-07-31 | IHUCOVID-32998 |
| 62 | A | 2020-03-27 | IHUCOVID-26655 |
| 62 | B | 2021-07-31 | IHUCOVID-26015 |
| 64 | A | 2020-10-23 | IHUCOVID-25934 |
| 65 | A | 2020-04-11 | IHUCOVID-16310 |
| 67 | A | 2021-03-19 | IHUCOVID-14544 |
| 67 | B | 2021-07-28 | IHUCOVID-23970 |
| 68 | B | 2021-07-28 | IHUCOVID-23998 |
| 69 | B | 2021-07-28 | IHUCOVID-23862 |
| 70 | A | 2020-09-14 | IHUCOVID-33687 |
| 70 | B | 2021-07-29 | IHUCOVID-24098 |
| 71 | A | 2020-08-19 | IHUCOVID-33685 |
| 71 | B | 2021-07-29 | IHUCOVID-24080 |
| 72 | B | 2021-07-28 | IHUCOVID-23949 |
| 73 | B | 2021-07-21 | IHUCOVID-20554 |
| 74 | A | 2021-02-08 | IHUCOVID-23580 |
| 76 | B | 2021-07-26 | IHUCOVID-21078 |
| 77 | A | 2020-09-03 | IHUCOVID-1602 |
| 79 | A | 2020-10-22 | IHUCOVID-38654 |
| 81 | B | 2021-07-26 | IHUCOVID-23932 |
| 85 | A | 2021-04-05 | IHUCOVID-23802 |
| 87 | A | 2020-09-24 | IHUCOVID-37090 |
| 88 | A | 2020-11-02 | IHUCOVID-24168 |
| 88 | B | 2021-07-23 | IHUCOVID-20936 |
| 91 | A | 2020-10-28 | IHUCOVID-20613 |
| 92 | A | 2020-10-14 | IHUCOVID-21357 |
| 92 | B | 2021-07-21 | IHUCOVID-20562 |
| 95 | B | 2021-07-23 | IHUCOVID-20995 |
| 96 | A | 2020-11-16 | IHUCOVID-14139 |
| 97 | B | 2021-07-23 | IHUCOVID-20940 |
| 100 | A | 2021-02-24 | IHUCOVID-25809 |
| 101 | A | 2020-09-07 | IHUCOVID-16368 |
| 101 | B | 2021-07-19 | IHUCOVID-18993 |
| 102 | B | 2021-07-19 | IHUCOVID-19000 |
| 103 | A | 2021-04-15 | IHUCOVID-18283 |
| 103 | B | 2021-07-17 | IHUCOVID-17544 |
| 104 | B | 2021-07-19 | IHUCOVID-17981 |
| 106 | A | 2020-09-01 | IHUCOVID-19878 |
| 107 | B | 2021-07-13 | IHUCOVID-16838 |
| 108 | B | 2021-07-07 | IHUCOVID-16707 |
| 109 | B | 2021-07-10 | IHUCOVID-16802 |
| 110 | A | 2020-08-27 | IHUCOVID-17552 |
| 110 | B | 2021-07-09 | IHUCOVID-16789 |
| 112 | A | 2021-03-15 | IHUCOVID-7727 |
| 113 | A | 2021-03-01 | IHUCOVID-10036 |
| 117 | B | 2021-03-30 | IHUCOVID-3989 |
| 119 | B | 2021-04-28 | IHUCOVID-8462 |
| 120 | B | 2020-08-07 | IHUCOVID-9905 |
| 123 | A | 2020-04-22 | IHUCOVID-9002 |
| 128 | A | 2020-10-02 | IHUCOVID-9903 |
| 129 | A | 2020-09-18 | IHUCOVID-1781 |
| 130 | B | 2021-05-06 | IHUCOVID-9618 |
| 135 | A | 2020-08-17 | IHUCOVID-1195 |
| 135 | B | 2020-11-28 | IHUCOVID-3080 |
| 138 | B | 2021-03-09 | IHUCOVID-6975 |
| 140 | B | 2021-03-31 | IHUCOVID-3980 |
| 141 | B | 2021-02-08 | IHUCOVID-8731 |
| 145 | A | 2020-03-19 | IHUCOVID-15328 |
| 147 | B | 2021-01-14 | IHUCOVID-8712 |
| 149 | B | 2021-04-12 | IHUCOVID-15554 |
| 151 | A | 2021-01-26 | IHUCOVID-11991 |
| 154 | A | 2020-07-29 | IHUCOVID-3950 |
| 154 | B | 2021-03-25 | IHUCOVID-3987 |
| 155 | A | 2020-09-02 | IHUCOVID-4137 |
| 156 | A | 2020-10-27 | IHUCOVID-3962 |
| 156 | B | 2021-03-15 | IHUCOVID-3985 |
| 157 | A | 2020-06-25 | IHUCOVID-0941 |
| 161 | A | 2020-08-12 | IHUCOVID-3951 |
| 162 | A | 2020-08-17 | IHUCOVID-1200 |
| 163 | A | 2020-08-11 | IHUCOVID-1469 |
| 163 | B | 2021-01-28 | IHUCOVID-8724 |
| 164 | A | 2020-08-10 | IHUCOVID-1443 |
| 165 | A | 2020-09-10 | IHUCOVID-3958 |
| 166 | B | 2020-08-19 | IHUCOVID-1347 |
| 171 | B | 2021-04-27 | IHUCOVID-5686 |
| 172 | B | 2020-12-21 | IHUCOVID-2971 |
| 179 | B | 2020-10-24 | IHUCOVID-9594 |
| 181 | A | 2020-09-03 | IHUCOVID-3957 |
| 181 | B | 2021-02-09 | IHUCOVID-8727 |
| 182 | A | 2020-05-03 | IHUCOVID-0788 |
| 183 | B | 2021-02-24 | IHUCOVID-8729 |
| 184 | A | 2020-11-03 | IHUCOVID-3965 |
| 185 | A | 2020-10-20 | IHUCOVID-2730 |
| 185 | B | 2021-02-04 | IHUCOVID-3238 |
| 186 | A | 2020-04-18 | IHUCOVID-15618 |
| 188 | A | 2020-04-25 | IHUCOVID-9004 |
| 188 | B | 2021-03-31 | IHUCOVID-3992 |
| 190 | B | 2021-02-05 | IHUCOVID-8726 |
| 192 | B | 2021-03-01 | IHUCOVID-8733 |
| 193 | A | 2020-03-27 | IHUCOVID-10598 |
| 197 | A | 2020-09-11 | IHUCOVID-11924 |
| 197 | B | 2021-05-26 | IHUCOVID-11842 |
| 198 | A | 2020-09-08 | IHUCOVID-5154 |
| 200 | B | 2021-01-14 | IHUCOVID-9596 |
| 202 | A | 2020-04-04 | IHUCOVID-0450 |
| 208 | B | 2021-03-19 | IHUCOVID-8741 |

Hospitalization

N = 19

Transfer to ICU

N = 5

Hospitalization

N = 13

Transfer to ICU

N = 6

Death

N = 2

209 re-infected patients

(Time between two episodes of infection ≥90 days AND clinical recovery with at least 1 qPCR negative after the first infection)

1^st^ infection

N = 209

159 primo-infected patients recruited during the same period of study and matched by age, sex, comorbidities, most frequent virus variants Delta (B.1.617.2)

20A.EU2 (B.1.160)

Alpha.V1 (B.1.1.7)

159 patients with clinical data and SARS-CoV-2 variants available

100 with 21A (Delta) (B.1.617.2)

34 with 20A.EU2 (B.1.160)

25 with 20I (Alpha.V1) (B.1.1.7)

Analysis of the severity of disease among 159 paired patients

2^nd^ infection

N = 209

Matched patients with age, sex, comorbidities, most frequent virus variants

**Supplementary Figure 1**: Flow-chart of patient selection


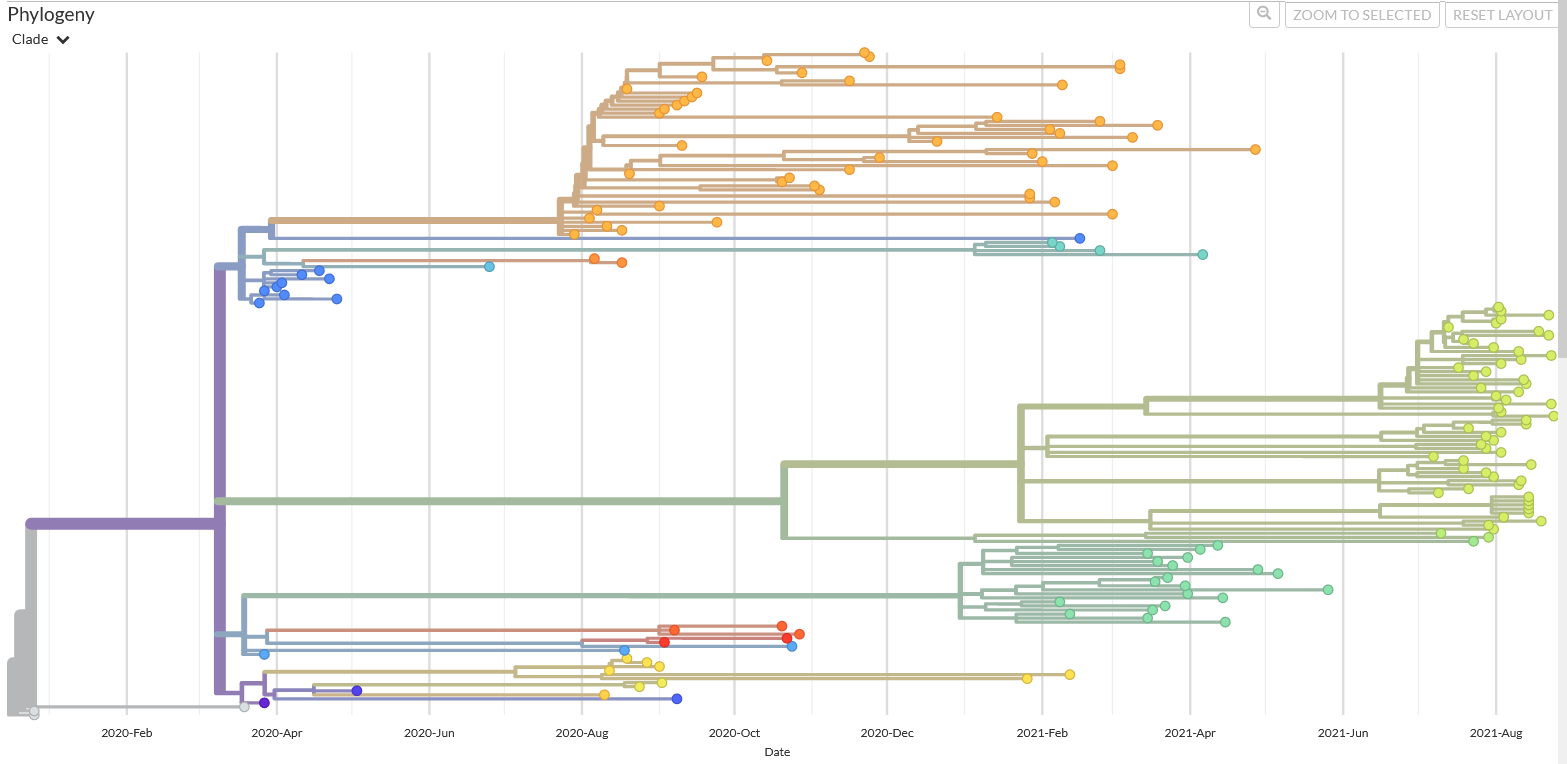

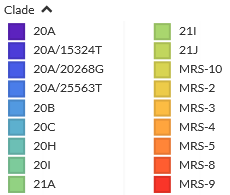


**Supplementary Figure 2.** Phylogenetic tree of SARS-CoV-2 genomes obtained from patients diagnosed with SARS-CoV-2 re-infection in our institute. Nextclade 19A and 19B genomes including from Wuhan-Hu-1 isolate (GenBank accession no. NC_045512.2) were used as reference sequences (indicated by gray lines and circles).
